# The evolution of zebrafish RAG2 protein is required for adapting to the elevated body temperature of the higher endothermic vertebrates

**DOI:** 10.1038/s41598-020-61019-w

**Published:** 2020-03-05

**Authors:** Ao Sun, Ke Xu, Haifeng Liu, Hua Li, Yaohuang Shi, Xiaoyan Zhu, Tao Liang, Xinyue Li, Xianxia Cao, Yanhong Ji, Taijiao Jiang, Chenqi Xu, Xiaolong Liu

**Affiliations:** 1 State Key Laboratory of Cell Biology, Shanghai Institute of Biochemistry and Cell Biology, Center for Excellence in Molecular Cell Science, Chinese Academy of Sciences, University of Chinese Academy of Sciences, Shanghai, 200031 China; 2 School of Life Science and Technology, ShanghaiTech University, Shanghai, 201210 China; 3 Department of Pathogenic Biology and Immunology, School of Basic Medical Sciences, Xi’an Jiaotong University Health Science Centre, Xi’an, Shaanxi 710061 China; 4 Center of System Medicine, Institute of Basic Medical Sciences, Chinese Academy of Medical Sciences & Peking Union Medical College, Beijing, 100005 China

**Keywords:** Evolution, VDJ recombination

## Abstract

The recombination activating gene (RAG or RAG1/RAG2 complex)-mediated adaptive immune system is a hallmark of jawed vertebrates. It has been reported that RAG originated in invertebrates. However, whether RAG further evolved once it arose in jawed vertebrates remains largely unknown. Here, we found that zebrafish RAG (zRAG) had a lower activity than mouse RAG (mRAG). Intriguingly, the attenuated stability of zebrafish RAG2 (zRAG2), but not zebrafish RAG1, caused the reduced V(D)J recombination efficiency compared to mRAG at 37 °C which are the body temperature of most endotherms except birds. Importantly, the lower temperature 28 °C, which is the best temperature for zebrafish growth, made the recombination efficiency of zRAG similar to that of mRAG by improving the stability of zRAG2. Consistent with the prementioned observation, the V(D)J recombination of *Rag2*^KI/KI^ mice, which zRAG2 was substituted for mRAG2, was also severely impaired. Unexpectedly, *Rag2*^KI/KI^ mice developed cachexia syndromes accompanied by premature death. Taken together, our findings illustrate that the evolution of zebrafish RAG2 protein is required for adapting to the elevated body temperature of the higher endothermic vertebrates.

## Introduction

Jawed vertebrates possess a diverse repertoire of T cell receptors (TCRs) and immunoglobulins (Igs) to specifically recognize and ultimately destroy unlimited numbers of foreign and lethal invaders or pathogens^1–3^. TCRs and Igs are assembled via the recombination of variable (V), diversity (D), and joining (J) gene segments^3–5^. The V(D)J recombination is a cut-and-paste reaction mediated by the RAG1/RAG2 complex (RAG) within specific genomic recombination signal sequence (RSS) sites^3,4,6^. Therefore, RAG triggers the TCR- and Ig-based adaptive immune system of jawed vertebrates to defense foreign invaders^7,8^.

In recent decades, many scientists have carefully explored the origin of RAG^8–10^. Our previous study has shown that bfRAG1L, an amphioxus RAG1-like DNA fragment, encodes a functional central domain of the vertebrate core RAG1^9^. Another study found that RAG1 originated from the Transib transposase and could independently mediate V(D)J recombination^10^. An interesting discovery was that of amphioxus ProtoRAG, which was demonstrated to be a cut-and-paste DNA transposon from amphioxus, placing the origination of RAG as far back as in basal chordates^8^.

Although the origin of RAG has been well studied, however, it has remained largely unknown whether RAG in jawed vertebrates from ectotherms to endotherms further evolved. The most evolutionarily ancient living jawed vertebrates are cartilaginous fish (e.g., horned sharks), which have been verified to have a complete set of RAGs, TCRs and Igs^2,11^. When lower jawed vertebrates evolved into higher jawed vertebrates, they faced not only the transition in themselves from ectothermy to endothermy but also the transition in environment from aquatic to terrestrial. This process occurred along with numerous environmental changes associated with air, water, temperature, humidity, ion concentrations, pathogenic microorganisms and body temperature (T_b_)^12,13^. For such great changes, we want to explore whether the adaptive immune system of jawed vertebrates, especially its hallmark RAG, evolved.

Before investigating whether there existed difference among jawed vertebrate RAG, we selected two species that are zebrafish (*Danio rerio*) and mice (*Mus musculus*) spanning the most vertebrate evolutionary history, and they are well characterized and widely used model animals^14–17^. Our results showed that the recombination efficiency of zebrafish RAG (zRAG) was lower than that of mouse RAG (mRAG) at 37 °C. Further research demonstrated that zRAG2, but not zRAG1 contributed to the lower recombination efficiency of zRAG compared to mRAG2 and it was due to its instability at 37 °C. Remarkably, when we moved the cells to the lower temperature 28 °C, which is the best temperature for the growth and staging of zebrafish, there was no difference between the zRAG2 and mRAG2 proteins regardless of their stability and enzymatic activity. In addition, to dissect what would have happened if RAG2 had not evolved from ectotherm RAG2, we generated the *Rag2*^KI/KI^ mice, in which zRAG2 was substituted for mRAG2. Consistent with previously mentioned observation, we found that the V(D)J recombination efficiency of *Rag2*^KI/KI^ mice was severely attenuated. To our surprise, *Rag2*^KI/KI^ mice developed cachexia syndromes accompanied by premature death. Collectively, our findings suggest that the evolution of zebrafish RAG2 protein is required for adapting to the elevated body temperature of the higher endothermic vertebrates.

## Results

### The recombination efficiency of zRAG is lower than that of mRAG at 37 °C

**Figure 1:**
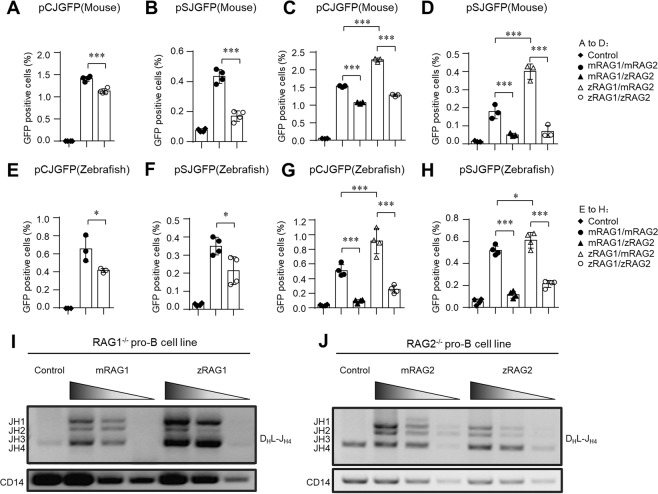
The recombination efficiency of zRAG is lower than that of mRAG at 37 °C. NIH3T3 cells were co-transfected with pCJGFP (mouse) (**A**,**C**), pSJGFP (mouse) (**B**,**D**), pCJGFP (zebrafish) (**E**,**G**) and pSJGFP (zebrafish) (**F**,**H**) and the indicated RAG1 and RAG2 using Lipo6000 transfection reagent. The GFP level was measured by flow cytometry to assess the recombination efficiency. The percentages of GFP positive cells were shown (the mean ± SD was calculated from triplicate experiments). (**I**,**J**) PCR analyses of the indicated D_H_J_H_ family rearrangements in RAG1-deficient pro-B cells (**I**) or RAG2-deficient pro-B cells (**J**). Input control: CD14 (bottom). PCR amplification was performed with fivefold serial dilutions of genomic DNA. The results are typical of three experiments. Bands related to rearrangements of various J_H_ segments are indicated on the left. The error bars indicate the SDs. The data are presented as the mean ± standard deviation. *P < 0.05, **P < 0.01 and ***P < 0.001 by Student’s t test.

Before investigating whether there existed evolution among jawed vertebrate RAGs, we first sought to determine whether there are functional differences among RAGs of jawed vertebrates. We selected two species whose evolution occurred four to five hundred of million years apart: the zebrafish and the mouse, which are a teleost fish and a mammal, respectively^14,17–19^. A GFP reporter recombination assay and the semi-quantitative PCR method were used to evaluate the recombination efficiency of NIH3T3 and pro-B cell lines, respectively^20–23^. The GFP reporter recombination assay for zRAG1/zRAG2 in the NIH3T3 and HEK-293T cell lines showed that there were fewer GFP positive cells that had undergone V(D)J recombination in the zRAG1/zRAG2 group than in the mRAG1/mRAG2 group regardless of whether the cells were co-transfected with coding joint (pCJGFP) or signal joint (pSJGFP) plasmids (Fig. 1A,B and Fig. S1A,B,E). Further research showed that recombination efficiency was decreased as long as zRAG2 participated in compared with mRAG2 when they combined with zRAG1 or mRAG1 respectively; the efficiencies were as follows: mRAG1zRAG2 < mRAG1mRAG2 and zRAG1zRAG2 < zRAG1mRAG2 (Fig. 1C,D and Fig. S1C–E). Intriguingly, RAG2, but not RAG1, contributed to the attenuated recombination efficiency. Given that RAG and 12/23RSS are core components of V(D)J recombination, we next examined whether the difference in recombination efficiency between zebrafish and mice was a result of their different 12/23RSSs. To investigate this possibility, we substituted zebrafish 12/23RSS for mouse 12/23RSS in the pCJGFP and pSJGFP plasmids. Interestingly, we still found that recombination efficiency was decreased when zRAG2, rather than mRAG2, was combined with zRAG1 or mRAG1; the efficiencies were as follows: mRAG1zRAG2 < mRAG1mRAG2 and, zRAG1zRAG2 < zRAG1mRAG2 **(**Fig. 1E–H**)**.

RAG is naturally expressed in B and T progenitor cells. Pro-B cells are B cell progenitors^24,25^. We wanted to further determine whether the decreased recombination efficiency was truly caused by zRAG2 in intact natural recombination machinery with the authentic recombination substrates within the genome and in the presence of the powerful non-homologous end joining (NHEJ) DNA repair pathway^3^. We performed a cell-based PCR assay to test the recombination efficiency in RAG1- or RAG2-deficient Abelson murine leukemia virus (A-MuLV)-transformed pro-B cell lines^20,21,26^. Consistent with our observations in the GFP reporter recombination assay with NIH3T3 cells, the levels of D_H_-J_H_ rearrangement were lower in RAG2-deficient pro-B cell lines when zRAG2 was combined with mRAG1 than when mRAG2 was combined with the same mRAG1 (Fig. 1I,J). These findings suggested that RAG2, but not RAG1, reduced the recombination efficiency of zebrafish in pro-B cells at 37 °C.

### The zRAG2 protein is unstable compared to the mRAG2 protein at 37 °C

**Figure 2:**
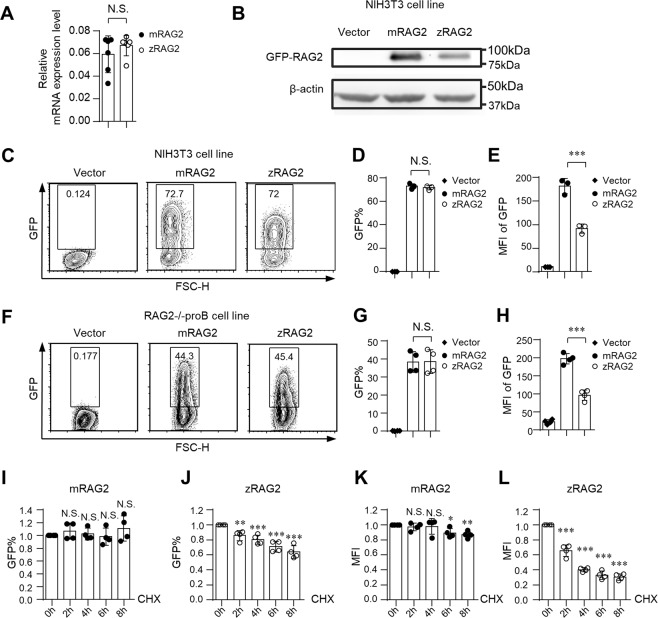
The zRAG2 protein is unstable compared to the mRAG2 protein at 37 °C. (**A**) Both zRAG2 and mRAG2 had the similar mRNA expression. The mRNA expression levels were normalized to β-actin expression. (**B**) The zRAG2 protein showed a lower expression level than the mRAG2 protein. β-actin served as a loading control for western blot analysis. (**C**–**E**) and (**F**–**H**) The zRAG2 protein showed an expression level similar to that of the mRAG2 protein, but its MFI was lower than that of mRAG2. zRAG2-GFP fusion protein and mRAG2-GFP fusion protein were introduced into NIH3T3 cells (**C**–**E**) or RAG2-deficient pro-B cells (**F**–**H**) by the retrovirus-mediated gene transfer method. After 36 h, GFP expression and intensity were analyzed by FACS. (**I**–**L**) The zRAG2 level was sensitive to proteasome degradation. RAG2-deficient pro-B cells were treated with CHX for 8 hours. GFP intensity was analyzed by FACS. (**I**,**J**) The GFP% of mRAG2 and zRAG2 was shown; (**K**,**L**) The MFI of mRAG2 and zRAG2 was shown. The error bars indicate the SDs. The data are presented as the mean ± standard deviation. *P < 0.05, **P < 0.01 and ***P < 0.001 by Student’s t test; N.S.: no significance.

We then questioned why RAG2 brought about the attenuated recombination efficiency of zebrafish. We first analyzed the mRNA expression quantity of mRAG2 and zRAG2. We found that there were no differences between them **(**Fig. 2A**)**. However, the western blot results showed that zRAG2 protein expression was lower than mRAG2 protein expression **(**Fig. 2B**)**. Next, we examined the biased findings based on single cell levels by FACS method^27^. Intriguingly, our FACS analysis showed that zRAG2 (zRAG2-GFP fusion protein) and mRAG2 (mRAG2-GFP fusion protein) exhibited similar transfection efficiencies which indicated that their amounts were not markedly different in the cells (Fig. 2C,D,F,G); however, there were distinct mean fluorescence intensity (MFI) differences in both cell lines: the MFI of zRAG2 were lower than the MFI of mRAG2 (Fig. 2C,E,F,H). In contrast, no difference was found between zRAG1 and mRAG1 with regard to either the percentage of GFP positivity or the MFI (Fig. S2A–F). Next, we further tested the protein stability in RAG2-deficient cell lines by quantifying the levels of the remaining zRAG2 and mRAG2 proteins at different time points after treating cells with the cycloheximide (CHX). The CHX chase experiment showed that the zRAG2 protein was initially unstable and had degraded by 2 h after the cells were placed in the 37 °C environments **(**Fig. 2J,L**)**. In contrast, mRAG2 hardly degraded and was stable throughout the experiment **(**Fig. 2I,K**)**. Additionally, we found that there was no difference between zRAG1 and mRAG1 with regard to the percentage of GFP positivity or the MFI when the cells were treated with CHX or CHX plus MG132 (Fig. S2G,H). This finding suggested that the zRAG2 protein was indeed unstable compared to the mRAG2 protein at 37 °C, whereas the stabilities of zRAG1 and mRAG1 did not differ.

Next, we wanted to know if other bony fish RAG2 proteins had the similar characterization. At first, we compared the amino acid sequence of three endotherm RAG2 (*Homo sapiens*, *Mus musculus and Rattus norvegicus*) and three ectotherm RAG2 proteins (*Oncorhynchus mykiss*, *Oreochromis niloticus* and *Danio rerio*) **(**Fig. S3**)**. We found that *Oreochromis niloticus* RAG2 (onRAG2), *Oncorhynchus mykiss* RAG2 (omRAG2) and zRAG2 proteins had sixty-four percent of identical amino acids **(**Fig. S3**)**. Therefore, we try to measure RAG2 protein stability and recombination activity of another two bony fish that are onRAG2 and omRAG2. We found that onRAG2 and omRAG2 proteins were also unstable like zRAG2 **(**Fig. S4B**)** while they had similar expression level **(**Fig. S4A**)**. The CHX chase experiment showed that onRAG2 and omRAG2 proteins like zRAG2 were initially unstable and had degraded by 2 h after the cells were placed in the 37 °C environments **(**Fig. S4C**)**. In contrast, mRAG2 hardly degraded and was stable throughout the experiment **(**Fig. S4C**)**. Furthermore, the recombination efficiency of onRAG2 and omRAG2 protein was also lower than mRAG2 **(**Fig. S4D**)**. These data, in combination with the characterization of zRAG2, indicated that RAG2 proteins in other teleost species were also unstable and had the lower recombination efficiency than mRAG2.

### The protein stability and recombination efficiency of zRAG2 are similar to those of mRAG2 at 28 °C

**Figure 3:**
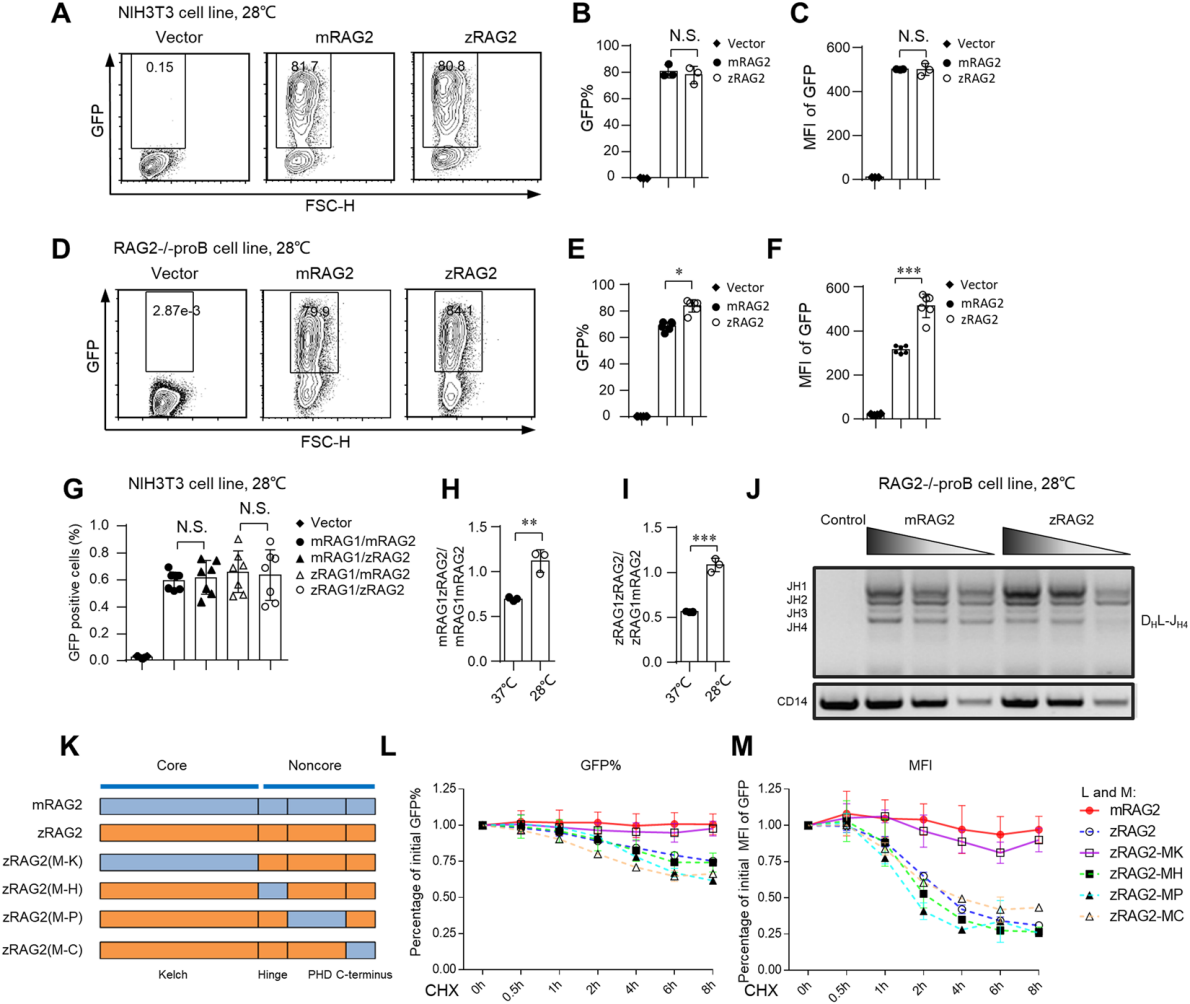
The protein stability and recombination efficiency of zRAG2 are similar to those of mRAG2 at 28 °C. (**A**–**F**) The zRAG2 protein showed an expression level similar to that of the mRAG2 protein, and its MFI was also similar to that of mRAG2 or even higher at 28 °C. zRAG2-GFP fusion protein and mRAG2-GFP fusion protein were introduced into NIH3T3 cells (**A**–**C**) or RAG2-deficient pro-B cells (**D**–**F**) by the retrovirus-mediated gene transfer method. The cells were first incubated for 36 h at 37 °C, and they were transferred to 28 °C after this time. After 48 h at 28 °C, GFP expression and intensity were analyzed by FACS. (**G**) NIH3T3 cells were transfected with pCJGFP using Lipo6000 transfection reagent. The GFP level was measured by flow cytometry to assess the recombination efficiency. The percentages of GFP-positive cells were shown (the means ± SDs were calculated from triplicate experiments). (**H**,**I**) The RAG1zRAG2/mRAG1mRAG2 and zRAG1zRAG2/zRAG1mRAG2 ratios were shown. (**J**) PCR analyses of the indicated D_H_J_H_ family rearrangements in RAG2-deficient pro-B cells at 28 °C. Input control: CD14 (bottom). PCR amplification was performed with fivefold serial dilutions of genomic DNA. The results are typical of three experiments. Bands related to rearrangements of various J_H_ segments are indicated on the left. (**K**–**M**) Different mouse RAG2 domains were substituted for the corresponding zRAG2 domains. GFP% (**L**) and MFI (**M**) are shown from 0 h to 8 h after treatment with the 20 μg/ml CHX. MK, mouse Kelch; MH, mouse hinge; MP, mouse PHD; MC, mouse C-terminus. The error bars indicate the SDs. The data are presented as the mean ± standard deviation. *P < 0.05, **P < 0.01 and ***P < 0.001 by Student’s t test; N.S.: no significance.

We then questioned why zRAG2 was more prone to instability than mRAG2. Given that the best temperature for the growth and staging of zebrafish is approximately 28 °C^28,29^, we sought to determine whether the stability and activity of zRAG2 was related to temperature. We first investigated zRAG2 protein stability after we moved both NIH3T3 cells and RAG2-deficient pro-B cells to a low temperature of 28 °C. To our surprise, FACS analysis showed that the MFI of zRAG2 in NIH3T3 cells was even higher than that of mRAG2 at 28 °C **(**Fig. 3A,C**)**, while the GFP expression levels had no difference between zRAG2 and mRAG2 48 hours (h) after the NIH3T3 cells had been moved from 37 °C to 28 °C **(**Fig. 3A,B). Next, we wondered what zRAG2 recombination efficiency would be since its stability was similar to that of mRAG2 at 28 °C. Interestingly, the GFP reporter recombination assay with NIH3T3 cells showed that the recombination efficiency of zRAG1zRAG2 and mRAG1zRAG2 was unexpectedly similar to that of mRAG1mRAG2 and zRAG1mRAG2 at 28 °C **(**Fig. 3G**)**. To further confirm that the role of zRAG2 in the recombination at 28 °C, we compared the mRAG1zRAG2/mRAG1mRAG2 and zRAG1zRAG2/zRAG1mRAG2 ratios at 37 °C and 28 °C. We found that the mRAG1zRAG2/mRAG1mRAG2 and zRAG1zRAG2/zRAG1mRAG2 ratios were twice as high at 28 °C as they were at 37 °C **(**Fig. 3H,I**)**. Moreover, similar observations regarding zRAG2 protein stability **(**Fig. 3D–F) and recombination efficiency were also made in RAG2-deficient pro-B cells at 28 °C **(**Fig. 3J**)**. These results suggested that the protein stability and recombination efficiency of zRAG2 displayed the similar to those of mRAG2 at 28 °C.

The RAG2 protein consists of Kelch (residues 1∼350), Hinge (residues ∼360–408), PHD (residues 414–487) and C-terminal domain (residues 488–527 or 488–530)^3,30–32^. Next, we wondered which domain contributed to the zRAG2 protein instability at 37 °C. We substituted the mRAG2 domain for the corresponding zRAG2 domains to form zRAG2-MK, zRAG2-MH, zRAG2-MP and zRAG2-MC (MK, mouse Kelch; MH, mouse hinge; MP, mouse PHD; and MC, mouse C-terminus) to investigate **(**Fig. 3K**)**. The CHX chase experiment results showed that the Kelch domain of mRAG2 could improve the MFI level of zRAG2 protein to an equal amount of mRAG2 while other domains including the Hinge, PHD and C-terminus had few improved effects on its stability **(**Fig. 3L,M**)**. These experiments indicated that the Kelch domain of zRAG2 mediated its instability and that the Kelch domain of mRAG2 could attenuate this instability.

### The V(D)J recombination of *Rag2*^KI/KI^ mice is severely impaired

**Figure 4:**
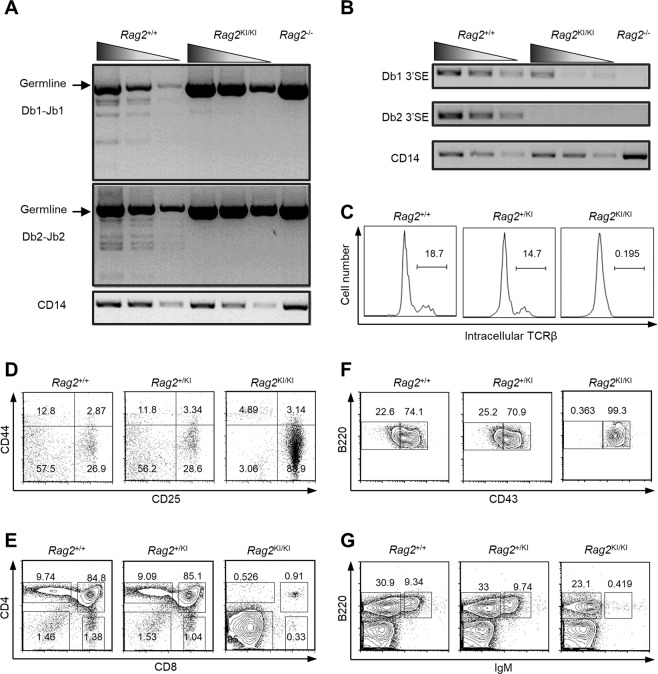
The V(D)J recombination of *Rag2*^KI/KI^ mice is severely impaired. Flow cytometric analysis of T and B cell development in *Rag2*^+/+^, *Rag2*^+/KI^ and *Rag2*^KI/KI^ mice. (**A**,**B**) TCRβ rearrangement was found to be decreased via semiquantitative PCR, which was used to detect D_β_-J_β_ rearrangement in DN3 thymocytes (CD4^−^CD8^−^CD44^−^CD25^+^ population) sorted from *Rag2*^KI/KI^ mice, their WT littermates and RAG2^−/−^ mice. Input control: CD14 (bottom). PCR amplification was performed with fivefold serial dilutions of genomic DNA. The results are representative of three independent experiments. (**C**) The intracellular TCRβ expression level in *Rag2*^KI/KI^ mice was largely diminished compared to that in *Rag2*^+/+^ or *Rag2*^KI/+^ mice. The TCRβ expression level was measured by flow cytometry. (**D**–**G**) The surface expression of CD4, CD8, B220, IgM, and CD43 in *Rag2*^KI/KI^ mice (mice with two zRAG2 knock-in alleles) and *Rag*2^+/+^ mice (WT littermates) was analyzed by flow cytometry. (**D**) CD44 and CD25 surface expression in DN cells of the thymus. (**E**) CD4 and CD8 surface expression on thymocytes. (**F**) IgM^−^ bone marrow cells were analyzed for surface expression of B220 and CD43. (**G**) The B220 and IgM surface expression on bone marrow cells. The results are representative of three independent experiments.

To investigate what the characteristics of mice would have been if zRAG2 had not evolved, we constructed a *Rag2*^KI/KI^ murine model, in which zRAG2 was substituted for mRAG2, via a homologous recombination method in ES cells **(**Fig. S5A–D**)**^33^. The V(D)J recombination efficiency of *Rag2*^KI/KI^ mice was severely attenuated compared to that of *Rag2*^+/+^ mice **(**Fig. 4A,B**)**, which was consistent with our observations in NIH3T3 and RAG2-deficient cells **(**Fig. 1A,B,J**)**. TCRβ intracellular staining further verified the extremely decreased V(D)J recombination efficiency of *Rag2*^KI/KI^ mice **(**Fig. 4C**)**. Additionally, analysis of the sequences of D_β_-J_β_ recombination products indicated that the signal joints of D_β1_-J_β1.1_ and D_δ_2-J_δ_1 recombination from the *Rag2*^KI/KI^ mice were similar to those from wild-type mice **(**Fig. S6A,B**)**. However, the impaired accuracy of V(D)J recombination in the *Rag2*^KI/KI^ mice was detected in D_β2_-J_β2.1_ recombination **(**Fig. S6C**)**. These results suggested that *Rag2*^KI/KI^ mice had impaired V(D)J recombination efficiency and accuracy. Consistently, *Rag2*^KI/KI^ mice showed severe combined immunodeficiency phenotypes because of impaired V(D)J recombination **(**Fig. 4D–G**)**. T cell development of *Rag2*^KI/KI^ mice was blocked in the early stages like *Rag2*^*−/−*^ mice which were blocked at the DN3 stage (CD4^−^ CD8^−^ double-negative stage) **(**Fig. 4D**)**. The percentage of DP cells (CD4^+^ CD8^+^ double positive cells) of the thymus was severely diminished (from 84.8% in wild-type mice to 0.9% in *Rag2*^KI/KI^ mice) **(**Fig. 4E**)**. Similar phenomena were also observed in B cells; the B cell development of *Rag2*^KI/KI^ mice was blocked at the pro-B stage, and the percentage of pre-B cells in bone marrow was also severely diminished (from 22.6% in wild-type mice to 0.3% in *Rag2*^KI/KI^ mice) **(**Fig. 4F**)**. The percentage of mature B cells was dramatically diminished from 9.3% in wild-type mice to 0.4% in *Rag2*^KI/KI^ mice **(**Fig. 4G**)**. These results indicated that the evolution of ectotherm RAG2 is required for adapting to the elevated body temperature of the higher endothermic vertebrates. Without this adaptation, endothermic jawed vertebrates would have an attenuated V(D)J recombination efficiency and lead to severe immunodeficiency.

### *Rag2*^KI/KI^ mice develop severe cachexia syndromes

**Figure 5:**
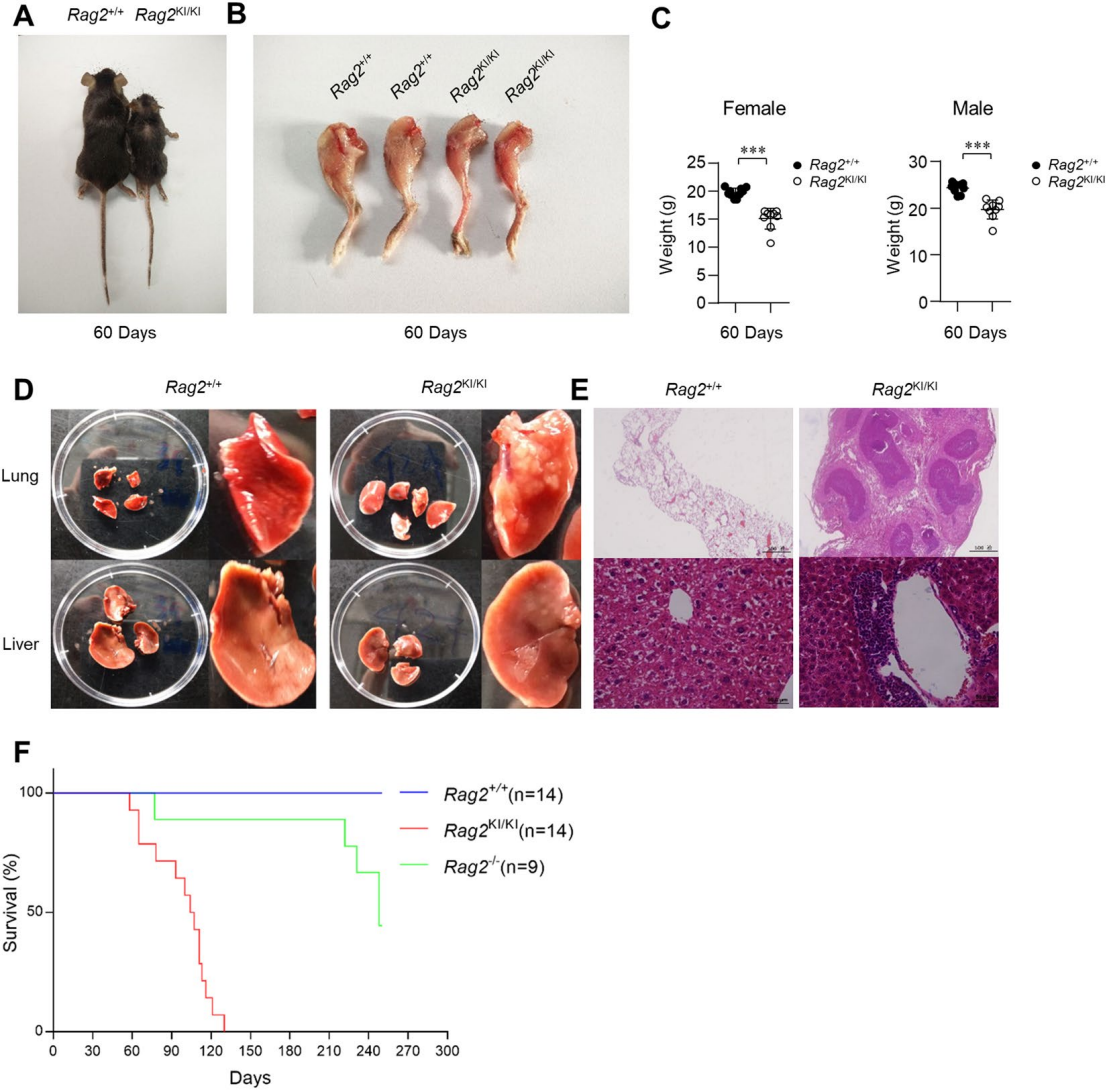
*Rag2*^KI/KI^ mice develop severe cachexia syndromes. (**A**) Representative photographs of 2 months old *Rag2*^KI/KI^ mice and wild-type *Rag2*^+/+^ littermates. (**B**) Skin was stripped from the hind limbs of 60 days old mice for gross comparisons of muscle mass. (**C**) Total body weight of 2 months old *Rag2*^+/+^ mice (n = 9) *Rag2*^KI/KI^ mice (n = 9). (**D**) Representative photographs of lungs and livers from 2–5 months old *Rag2*^KI/KI^ mice and wild-type *Rag2*^+/+^ littermates. (**E**) Representative H&E staining of lung and liver sections at 2–5 months old *Rag2*^KI/KI^ mice and wild-type *Rag2*^+/+^ littermates. (**F**) Kaplan–Meier curves of *Rag2*^+/+^ mice, *Rag2*^KI/KI^ mice and *Rag2*^*−*/*−*^ mice (*Rag2*^+/+^ mice, n = 14; *Rag2*^KI/KI^ mice, n = 14; *Rag2*^*−*/*−*^ mice, n = 9). Chi square log-rank test was used for statistical analysis. The results are representative of three independent experiments. The error bars indicate the SDs. The data are presented as the mean ± standard deviation. *P < 0.05, **P < 0.01 and ***P < 0.001 by Student’s t test.

To our surprise, *Rag2*^KI/KI^ mice unexpectedly not just had a severely impaired V(D)J recombination efficiency, but developed severe cachexia syndromes that were characterized by smaller body size **(**Fig. 5A**)**, skeletal muscle wasting **(**Fig. 5B**)**, significant weight loss **(**Fig. 5C**)**, systematic immune infiltration **(**Fig. 5D,E**)**. More interestingly, *Rag2*^KI/KI^ mice had a shortened lifespan even than *Rag2*^*−*/*−*^ mice **(**Fig. 5F**)**.

**Figure 6:**
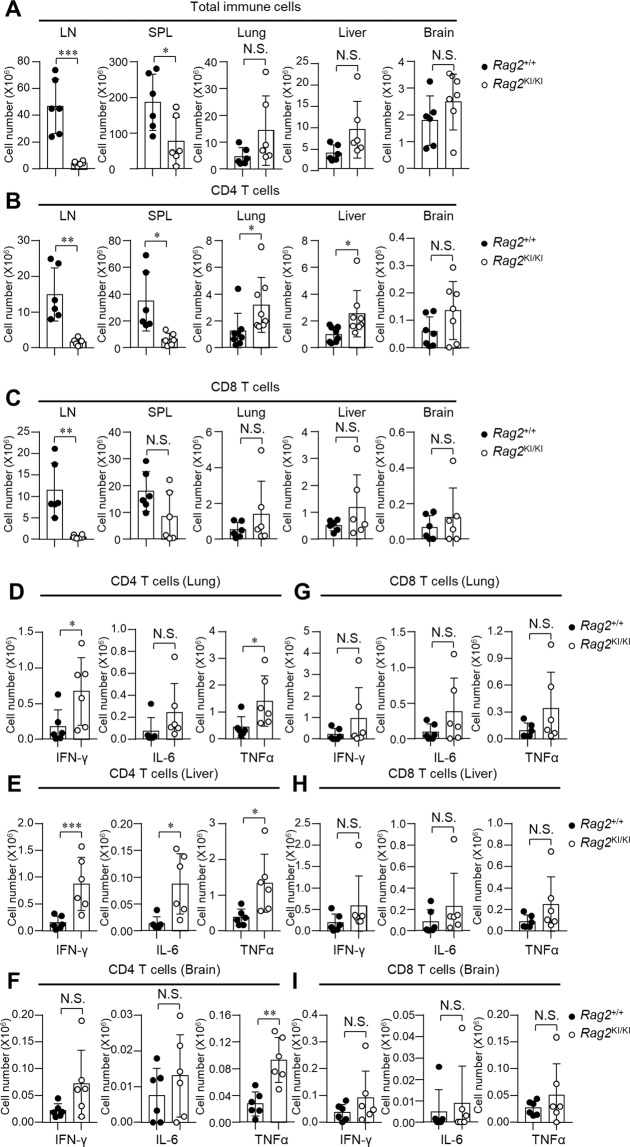
T cells that produced cachexia cytokines of *Rag2*^KI/KI^ mice are disordered. (**A**) Total immune cells of lymph nodes (LN), spleen (SPL), lung, liver and brain from *Rag2*^+/+^ and *Rag2*^KI/KI^ mice. *Rag2*^+/+^ mice (n = 6); *Rag2*^KI/KI^ mice (n = 6). (**B**) The absolute CD4 T cells of LN, SPL, lung, liver and brain from *Rag2*^+/+^ and *Rag2*^KI/KI^ mice. *Rag2*^+/+^ mice (n = 6); *Rag2*^KI/KI^ mice (n = 6). (**D**–**F**) The disorder of IFN-γ, IL-6 and TNFα of CD4 T cells in lung (**D**), liver (**E**) and brain (**F**) from *Rag2*^+/+^ and *Rag2*^KI/KI^ mice was analyzed. *Rag2*^+/+^ mice (n = 6); *Rag2*^KI/KI^ mice (n = 6). (**C**) The absolute CD8 T cells of LN, SPL, lung, liver and brain from *Rag2*^+/+^ and *Rag2*^KI/KI^ mice. *Rag2*^+/+^ mice (n = 6); *Rag2*^KI/KI^ mice (n = 6). (**G**–**I**) The disorder of IFN-γ, IL-6 and TNFα of CD8 T cells in lung (**G**), liver (**H**) and brain (**I**) from *Rag2*^+/+^ and *Rag2*^KI/KI^ mice was analyzed. *Rag2*^+/+^ mice (n = 6); *Rag2*^KI/KI^ mice (n = 6). All cytokines were detected by flow cytometric analysis after *in vitro* stimulation with PMA (phorbol 12-myristate 13-acetate) and ionomycin in the presence of brefeldin A for 4 h. The error bars indicate the SDs. The data are presented as the mean ± standard deviation. *P < 0.05, **P < 0.01 and ***P < 0.001 by Student’s t test; N.S.: no significance.

Chronic inflammation is one of the major drivers of cachexia^34,35^. It have been identified that four pro-inflammatory cytokines including tumor necrosis factor alpha (TNF-α, initially named cachectin), interferon-γ (IFN-γ), interleukin-6 (IL-6) and interleukin-1 (IL-1) contributed to the cachexia syndrome^34,36^. In the four cachexia cytokines, T cell expressed IFN-γ, TNF-α and IL-6^37–41^. Interestingly, we found that CD4 T cells and CD8 T cells from *Rag2*^KI/KI^ mice were highly activated characterized by the increased CD69^high^ cells **(**Fig. S7A,B**)** and CD44^high^ CD62^low^ cells **(**Fig. S7C,D**)** in the lymph nodes, spleen, lung, liver and brain. To our surprise, we found that the absolute cell numbers of total immune cells from peripheral immune organs of *Rag2*^KI/KI^ mice was dramatically decreased while the cell numbers of total immune cells from non-immune organs of *Rag2*^KI/KI^ mice was no less **(**Fig. 6A**)**. Further research showed that CD4 T cells were markedly increased **(**Fig. 6B**)** and they ectopically produced different cachexia cytokines in different non-immune organs **(**Fig. 6D–F and Fig. S8A–C**)**. In the lung, IFN-γ and TNF-α produced by CD4 T cells of *Rag2*^KI/KI^ mice were increased compared to *Rag2*^+/+^ mice **(**Fig. 6D and Fig. S8A**)**. In the liver, IFN-γ, TNF-α and IL-6 produced by CD4 T cells of *Rag2*^KI/KI^ mice were increased compared to *Rag2*^+/+^ mice **(**Fig. 6E and Fig. S8B**)**. In the brain, TNF-α produced by CD4 T cells of *Rag2*^KI/KI^ mice were specially increased compared to *Rag2*^+/+^ mice **(**Fig. 6F and Fig. S8C**)**. There was no increase found in CD8 T cells from the three non-immune organs (lung, liver and brain) of *Rag2*^KI/KI^ mice compared to *Rag2*^+/+^ mice **(**Fig. 6C**)**, and the absolute numbers of CD8 T cells that produced cachexia cytokines of *Rag2*^KI/KI^ mice were also no increase compared to *Rag2*^+/+^ mice **(**Fig. 6G–I**)** in spite of the dramatically increased percentage of these cachexia cytokines produced by CD8 T cells. **(**Fig. S8D–F**)**. Our results identified that *Rag2*^KI/KI^ mice developed severe cachexia syndromes from individual phenotype to molecular and cell levels. These results suggested that the lower ectothermic vertebrate RAG2 might further evolve to adapt to the elevated body temperature of the higher endothermic vertebrates in the evolution of jawed vertebrates. Otherwise, endothermic jawed vertebrates would have not only had weaker recombination efficiency and immunodeficiency but also developed severe cachexia syndromes.

## Discussion

Although the origination of RAG in invertebrates has been extensively studied, the evolution of RAG in jawed vertebrates remains largely unknown. Our findings suggest that the evolution of zebrafish RAG2 protein is required for adapting to the elevated body temperature of the higher endothermic vertebrates. Importantly, zRAG2, but not zRAG1, which possesses endonuclease activity, is responsible for the reduced recombination efficiency of zRAG compared to mRAG at 37 °C. If the lower ectotherm RAG2 such as zRAG2 did not evolve, endothermic jawed vertebrates such as mice would have not only had weaker recombination efficiency and immunodeficiency but also developed severe cachexia syndromes.

Protein adaptation is essential for organismal evolution^42,43^. Allosteric regulation is a general and widespread evolutionary adaptation mechanism that fine-tunes biological function without changing fundamental structures^43–45^. RAG recombinase as an allosteric protein is composed of a heterotetrameric complex including two subunits each of RAG1 and RAG2^3,30,31^. RAG1, as an endonuclease, plays direct roles in both RSS binding and DNA cleavage, and is the actual workhorse in the process of V(D)J recombination^3,10,46,47^. In contrast, RAG2 is a vital allosteric factor whose principal function seems to interact with RAG1 and activate the enzymatic activity of RAG1 endonuclease^3,47,48^. Allosteric activators and cofactors gain their complementary regulatory properties simultaneously for specific functions, and the cofactors offer an advantage under evolutionary selective pressure^43,49,50^. This strategy for evolution is called conditional neutrality^43^. The mutation of cofactors may be regarded as a better strategy for adaption under evolutionary pressure than the mutation activator^43^. Thus, in the machinery of allostery, RAG1 can be regarded as the activator whereas RAG2 can be referred to as the cofactor. It has been reported that RAG2 can alter the conformation of RAG1 and stabilize interactions of RAG1 with the heptamer^47^. Similar to other allosteric proteins, RAG2 may have adapted during evolution via mutation. Importantly, the RAG2 Kelch domain is located at the interface of binding between RAG1 and RAG2. Mutations in the RAG2 Kelch domain block initiation of V(D)J recombination and lead to severe combined immunodeficiency (SCID) or Omenn syndrome (OS) immunodeficiency^51,52^. Hence, RAG2 mutations, particularly those in the Kelch domain, can likely be regarded as candidates driving RAG adaptation during the evolution of jawed vertebrates from ectotherms to endotherms without abrogating the rearrangement function of RAG1, consistent with the property of conditional neutrality.

Zebrafish mainly live in freshwater streams and rivers of northeastern India. Due to monsoon climate in their native geographic range, zebrafish have adapted to a broad range of temperatures, including temperatures from 12 °C to 39 °C^53–55^. Our results suggest that zRAG1/mRAG2 performs a fiercer recombination than mRAG1/mRAG2 and zRAG1/zRAG2. It has been reported that RAG endonuclease is a potent threat to genome stability because the lymphocyte genome owns thousands of RSS sites that can be recognized and bound by RAG^25^. Epistasis is the primary factor in molecular evolution^56–59^. For keeping constant metabolic capacity, many ectothermic vertebrate enzyme activities are upregulated or downregulated in their fluctuating thermal environments^60^. Given that zRAG can well perform the V(D)J recombination at low environmental temperature 28 °C, we speculate that zebrafish may constrain zRAG1 endonuclease hyperactivity at high environmental temperature 37 °C via zRAG2 stability-mediated antagonistic epistasis to constrain the potential threat of RAG1 endonuclease for the genome stability.

The process of evolution from ectotherms to endotherms was one of the most prominent events in vertebrate evolution^60^. These dramatic transitions in the organisms themselves brought many challenges to the ectotherms that would ultimately achieve high and stable body temperatures of 37–42 °C^61^. Temperature profoundly affects every physiological function (e.g., metabolism, locomotion, and fecundity)^62,63^. However, details of the evolution of the adaptive immune system in this progress, especially at the molecular level, remain unclear. Our study suggests that the core component RAG of the adaptive immune system, especially RAG2, is indeed different between ectotherms and endotherms. It has been reported that RAG may have originated in the invertebrate amphioxus^8,9^. However, the amphioxus such as *Asymmetron lucayanum* lives in seawater with annual temperatures ranging from 23 °C to 29 °C (monthly means) which are lower than 37 °C^64^. It is reasonable to assume that RAG had no necessity or pressure to adapt to high temperatures in the beginning. Considering this assumption, we propose that the adaptive immune system of jawed vertebrates represented by RAG2 might have further evolved to adapt to a high and stable Tb during the evolution from ectotherms to endotherms.

It has been reported that the mutations in the positions V8, F62, T77, C446 and C478 from RAG2 would lead to structural destabilization of the RAG2 protein or RAG1/2 complex^65–67^. We compared the amino acid sequence of endotherm RAG2 and ectotherm RAG2 proteins. We found that V8 and T77 were not conserved between ectotherms and endotherms while the C446 and C478Y from RAG2 were conserved and F62 except omRAG2 was conserved between ectotherms and endotherms. Therefore, the phenotype of the knock-in mouse may be somewhat because of the instability of zRAG2/mRAG1 protein complex. More importantly, the cachexia syndromes were not found in our previous study on *Rag2*^C478Y^ mice that the V(D)J recombination efficiency of *Rag2*^C478Y^ protein was also dramatically attenuated because of its instability at 37 °C^67^. Given that zRAG2 is a genuine existed RAG2, not a mRAG2 mutant, we speculate that zRAG2 may have other functions besides the V(D)J recombination at 37 °C that do not function at 28 °C considering the products of V(D)J recombination mediated by zRAG2. Specially, why zRAG2 results in the severe cachexia syndromes of *Rag2*^KI/KI^ mice remains further research.

## Materials and Methods

### Mice

PGK-Cre transgenic mice were kindly provided by Dr. X. Wu (Fudan University, Shanghai, China). To generate DaR2 knock-in mice, targeting vectors containing DaR2, a diphtheria toxin A (DTA) negative selection cassette, and a PGK-neo cassette were used for the electroporation of embryonic stem (ES) cells. The targeted ES cell clones were kindly provided by the Stem Cell Bank, Chinese Academy of Sciences. These clones were microinjected into C57BL/6 blastocysts using a PiezoXpert (Eppendorf) to generate chimeric mice. Male chimeras were bred to C57BL/6 female mice, and the heterozygous progeny were crossed with PGK-Cre mice to remove the floxed PGK-Neo cassette. All mice were maintained in specific pathogen-free facilities. All mice were genotyped using PCR analysis before experimentation, and all animal experiments were approved by the Institutional Animal Care and Use Committee of the Shanghai Institutes for Biological Sciences of the Chinese Academy of Sciences. We confirmed that all experiments were performed in accordance with relevant guidelines and regulations.

### Plasmids

pEBG-RAG1 (mouse) and pEBG-RAG2 (mouse), which have been previously described, were used for recombination assays in NIH3T3 and HEK-293T cells. DNAs encoding the zRAG2, mRAG2, omRAG2 and onRAG2 genes were synthesized by Sangon Biotech. Then, the synthesized RAG2 genes were cloned into pEBG and pMX plasmid vectors. For RAG2 or RAG1 protein stability assays in NIH3T3 and RAG1^*−*/*−*^ or RAG2^*−*/*−*^ pro-B cells, pMX-GFP-mRAG2 and pMX-GFP-mRAG1 vectors were used; these vectors have been previously described^67,68^.

### Cell culture and transfection

Abelson-transformed Rag2^*−*/*−*^ tg.bcl2 pro- B cells were maintained in complete RPMI 1640 medium (Hyclone). Retroviral transfection was performed as described previously. Briefly, Plat-E cells were transfected with 2.5 μg of GFP-tagged RAG2 in a pMX-flag vector. The supernatant was collected 48 h post transfection, polybrene was added at a concentration of 5 μg/mL, and Rag2^−/−^ pro-B cells were resuspended at 0.6 million cells/mL. The transduction period lasted for 12 h, and the cells were analyzed after 48 h by flow cytometry.

### Antibodies and reagents

Anti-CD4 (RM4-5), anti-CD8a (53–6.7), anti-CD44 (IM-7), anti-CD25 (PC61), anti-Cd11b (M1/70), anti-TER-119 (TER-119), anti-IgM (R6-60.2), anti-TCRβ (H57-597), anti-CD62L (MEL-14), anti-CD69 (H1.2F3) anti-IL-6 (MP5-20F3) and TNF-α (MP6-XT22) were purchased from BD Pharmingen. Anti-B220 (RA3-6B2) and anti-CD3 (145-2C11), anti-IFN-γ (XMG1.2) were purchased from BioLegend. Anti-CD43 (GL3) was purchased from eBioscience (eBioR2/60). PMA and ionomycin were purchased from Merck. Cycloheximide was purchased from Sigma. MG-132 was obtained from Merck. Taq Plus Master Mix for genotyping was from Vazyme Biotech. Chloroquine was obtained from MCE. Collagenase IV was obtained from Sigma. DNase I was obtained from Shanghai Sanjie.

### Cell preparation, staining, and purification

Single cell from thymus, lymph nodes, spleen, liver, lung and brain suspensions were prepared and surface-stained as described previously^9,69–71^. Briefly, the thymus and lymph nodes were dissected from mice, and single-cell suspensions were prepared by gently teasing the tissue with forceps and then passing it through nylon filters. The liver and brain were dissected from the body, cut into pieces, minced in PBS buffer and thereafter filtered through a 40 μm cell strainer. The lungs were cut into pieces and incubated with shaking (200 rpm) at 37 °C for 1.5 h in RPMI medium (Gibco) containing 5% FBS (Gibco), 160 μg/ml collagenase IV (Sigma) and 0.2 μg/ml DNase I (Shanghai Sanjie). Leukocytes were isolated from the liver and brain cell suspensions or digested lungs by density fractionation using discontinuous 40–70% (vol/vol) Percoll (GE Healthcare) gradients. The cells were distributed in 5 mL polystyrene round-bottom tubes (Corning, Inc.) and stained for 40 min at 4 °C with the indicated antibodies. Cell fluorescence was observed using a two-laser FACSCalibur (BD Biosciences), a five-laser BD LSRFortessa (BD Biosciences) and a three-laser CytoFLEX (Beckman) flow cytometers. The data were analyzed with FlowJo software (TreeStar, Inc., Olten, Switzerland). Subsets of thymocytes were sorted using a FACSAria II flow cytometer (BD Biosciences).

### Intracellular staining

Intracellular staining was performed as described previously^70,71^. For intracellular staining, cells were stimulated with 50 ng/ml PMA (phorbol 12-myristate 13-acetate), 1 μg/ml inomycin, and 1 μg/ml brefeldin A for 4 h. After 4 h, these cells were fixed with 2% PFA, permeabilized with Perm cell permeabilization kit (eBiosciences), and stained with antibodies specific to intracellular cytokines. For intracellular staining of TCRβ, cells were fixed with 2% PFA, permeabilized with Perm cell permeabilization kit (eBiosciences), and stained with antibodies specific to TCRβ.

### PCR analysis of Tcrb, Tcrd and IgH recombination products

Genomic DNA was prepared from bone marrow pro-B (B220^+^IgM^−^CD43^high^) and DN3 cells (CD4^−^CD8^−^CD44^−^CD25^+^) that were sorted with a FACSAria II (BD Biosciences). For RAG1-deficient or RAG2-deficient pro-B cell lines, the genomic DNA was directly extracted^9,68,69^. Then, fivefold serial dilutions of the genomic DNA (200 ng, 40 ng, and 8 ng) were used to detect TCRβ, TCRγδ and IgH recombination by PCR, and CD14 was used as an internal control^9,68,69^. The primers for D_β_1J_β_1, D_β_2J_β_2, D_δ_2J_δ_2, D_H_J_H_ and CD14 were previously described^9,26,68,69^. The following numbers of PCR cycles were used to detect the recombinant products: for D_β_1J_β_1, D_β_2J_β_2, D_δ_2J_δ_2 and D_H_J_H_ coding joints, 28 cycles; for CD14, 26 cycles; and for D_δ_1J_δ_1 SJs, 35 cycles^9,26,68,69^. The PCR cycles for these analyses were as follows: 30 s at 95 °C, 30 s at 60 °C and 2.5 min, 30 s at 72 °C. The PCR products were separated on 1.5% agarose gels and then subjected to TA cloning (TaKaRa). The resulting plasmids were transformed into *E. coli* DH5α cells for sequence analysis.

### RNA isolation and quantitative real-time PCR analysis

Total RNA was extracted from FACS-sorted pro-B (B220^+^IgM^−^CD43^high^) and DN3 cells (CD4^−^CD8^−^CD44^−^CD25^+^) using TRIzol reagent (Invitrogen), and then the RNA was reverse transcribed with a HiScript II Q RT SuperMix for qPCR Kit (Vazyme). Real-time PCR was performed with SYBR Green Realtime PCR Master Mix (Toyobo) on a Bio-Rad CFX96 3.1 PCR system. β-actin was used as an internal control for RT-PCR. The primer pairs for the genes examined were as follows: mRAG2, 5′-CCAGGAGACAATAAGCAGGCTATGTCAG-3′ and 5′-CATCAAAACTGGTTGCTTCAGCACTG-3′; zRAG2, 5′- TGAGACTCAGAAGCGCATGG-3′ and 5′-ACCAAGTACGACTGTGGCTG-3′; and β-actin, 5′-GACGGCCAGGTCATCACTATTG-3′ and 5′-AGGAAGGCTGGAAAAGAGCC-3′.

### Recombination assay of plasmid substrate in NIH3T3 cells and HEK-293T cells

Recombinant pCJGFP (700 ng) or pSJGFP (700 ng) fluorescent substrate plasmids were cotransfected along with pEBG-RAG1 (1500 ng) and pEBG-RAG2 (1500 ng) or an equal amount of empty vector into HEK-293T cells by calcium phosphate precipitation or into NIH3T3 cells with Lipo6000 reagent (Beyotime)^9,21,23,69,72^. After 48 h, the recombination products were detected based on the percentage of cell GFP expression with a FACSCalibur (BD) machine. Then, the data were analyzed with FlowJo.

Recombinant pJH289 (700 ng) or pJH290 (700 ng) substrate plasmids were cotransfected with pEBG-RAG1 (1500 ng) and pEBG-RAG2 (1500 ng) or an equal amount of empty vector into HEK-293T cells by calcium phosphate precipitation^9,68,72^. After 48 h, the transfected DNA was extracted and purified with a GeneJET™ Genomic DNA Purification Kit. The assays for detection and analysis were performed as previously described^68^. The following primer pairs were used: for pJH289-SJ and pJH290-CJ, 5′-CCCCAGGCTTTACACTTT-3′ and 5′-CCGTCTTTCATTGCCATAC-3′; for pJH289 and pJH290 input, 5′-TCTTTATAGTCCTGTCGGGTTT-3′ and 5′-GCGGTAATACGGTTATCCAC-3′.

### Immunoblot analysis

Cells were lysed on ice for 30 min in RIPA lysis buffer (Beyotime) with a protease inhibitor cocktail (Cell Signaling Technology, CST), and the lysates were cleared by centrifugation. The protein extracts were separated by SDS-PAGE and analyzed by immunoblotting with the indicated Abs and HRP-conjugated secondary Abs (Santa Cruz Biotechnology). Antibodies for β-actin were used at a dilution of 1:5000 (Multisciences (Lianke) Biotech, Co., Ltd.), and antibodies for GFP (sc-9996; Santa Cruz Biotechnology) were used at a dilution of 1:1000. The bands on the blots were visualized with an ECL kit (34096; Thermo).

### Histology staining

Perfused lungs and livers from killed mice were dissected, fixed in 4% (v/v) buffered neutral formalin and embedded in paraffin. Five-micron tissue sections were stained with hematoxylin and eosin. All images were acquired with an Olympus BX51 microscope and DP71 camera was used to acquire (Olympus).

### Statistical analysis

For comparison between groups, all data are expressed as the mean ± SD. The results were analyzed by Student’s *t*-test. A P value less than 0.05 was considered significant.

## Supplementary information

Supplementary information

Supplementary information 2
