# Supplementary material for: The evolution of zebrafish RAG2 protein is required for adapting to the elevated body temperature of the higher endothermic vertebrates
Source: Sci Rep. 2020 Mar 5;10:4126. doi: 10.1038/s41598-020-61019-w (PMC7057966; doi:10.1038/s41598-020-61019-w)
Supplement: Supplementary file 1 — Supplementary information [file 41598_2020_61019_MOESM1_ESM.pdf]

Supplementary information

The evolution of zebrafish RAG2 protein is required for adapting to the elevated body temperature of the higher endothermic vertebrates

Ao Sun[1,+], Ke Xu[1,+], Haifeng Liu[1], Hua Li[1], Yaohuang Shi[1,2], Xiaoyan Zhu[1], Tao Liang[1], Xinyue Li[1], Xianxia Cao[1,2], Yanhong Ji[3], Taijiao Jiang[4], Chenqi Xu[1] and Xiaolong Liu[1,2,*]

[1]State Key Laboratory of Cell Biology, Shanghai Institute of Biochemistry and Cell Biology, Center for Excellence in Molecular Cell Science, Chinese Academy of Sciences, Shanghai 200031; University of Chinese Academy of Sciences, China

[2]School of Life Science and Technology, ShanghaiTech University, Shanghai 201210, China;

[3]Department of Pathogenic Biology and Immunology, School of Basic Medical Sciences, Xi'an Jiaotong University Health Science Centre, Xi'an, Shaanxi, 710061, China;

[4]Center of System Medicine, Institute of Basic Medical Sciences, Chinese Academy of Medical Sciences & Peking Union Medical College, Beijing, 100005, China

[*]Correspondence should be addressed to X.L. (liux@sibcb.ac.cn)

[+]A.S. and K.X. contributed equally to this work.

Keywords: RAG2, stability, temperature, evolution, jawed vertebrates, cachexia

Supplementary information

Figure S1. zRAG2 has lower recombination efficiency compared to mRAG2 in HEK-293T cells

Figure S2. Both mRAG1 and zRAG1 have a similar stability.

Figure S3. Comparison of amino acid sequence of ectotherms and endotherms.

Figure S4. Comparison of RAG2 protein stability and recombination efficiency among three bony fish and mouse.

Figure S5. Generation of *Rag2*$^{KI/KI}$ mice.

Figure S6. Comparison of recombination products between *Rag2*$^{KI/KI}$ and *Rag2*$^{+/+}$ mice.

Figure S7. T cells of *Rag2*$^{KI/KI}$ mice exhibit a hyperactivated phenotype.

Figure S8. The increased percentage of cachexia cytokines IFN-γ, IL-6 and TNFα produced by CD4 and CD8 T cells of *Rag2*$^{KI/KI}$ mice.

Figure S1

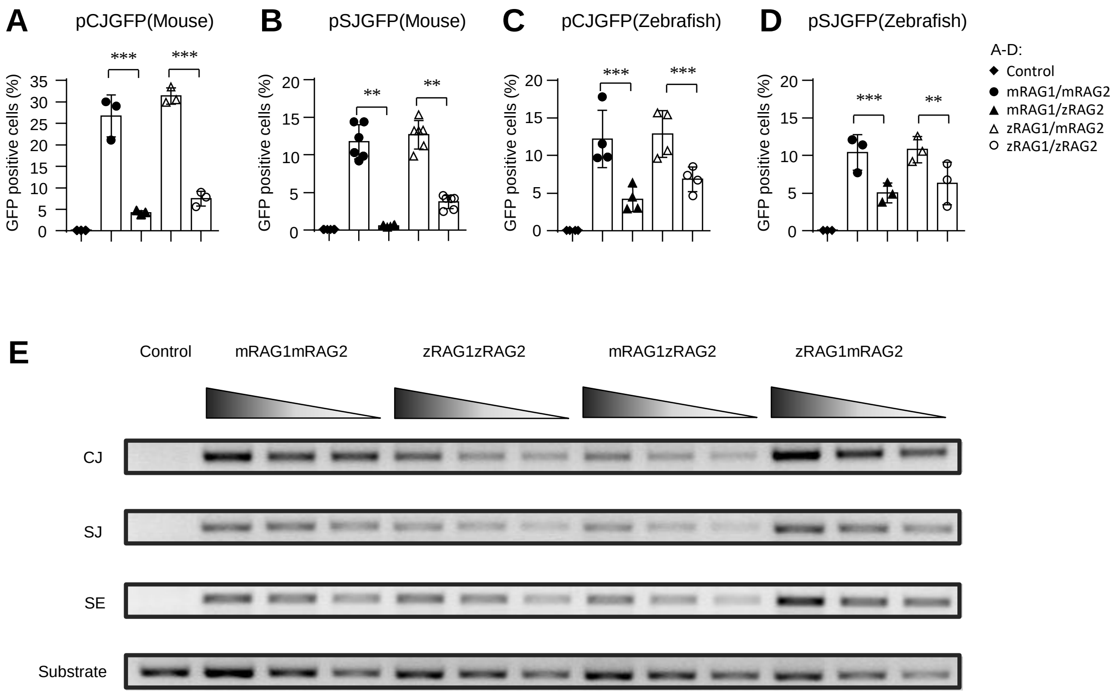

Figure S1. zRAG2 has lower recombination efficiency compared to mRAG2 in HEK-293T cells. HEK-293T cells were co-transfected with the pCJGFP (mouse) (A), pSJGFP (mouse) (B), pCJGFP (zebrafish) (C) and pSJGFP (zebrafish) (D) and indicated RAG1and RAG2 using calcium phosphate. The GFP positive cells which represent the recombination efficiency was measured by flow cytometry. The percentages of GFP positive cells are shown (the means ± SD is calculated from at least triplicate experiments). Error bars indicate the SDs. The data are means ± standard deviation: **P < 0.01, ***P < 0.001 by Student's t test. (E) The recombination products CJ (coding joint), SJ (signal joint) and SE (signal end) of pJH290-CJ (mouse) and pJH289-SJ (mouse) in HEK-293T cells were analyzed by PCR.

Figure S2

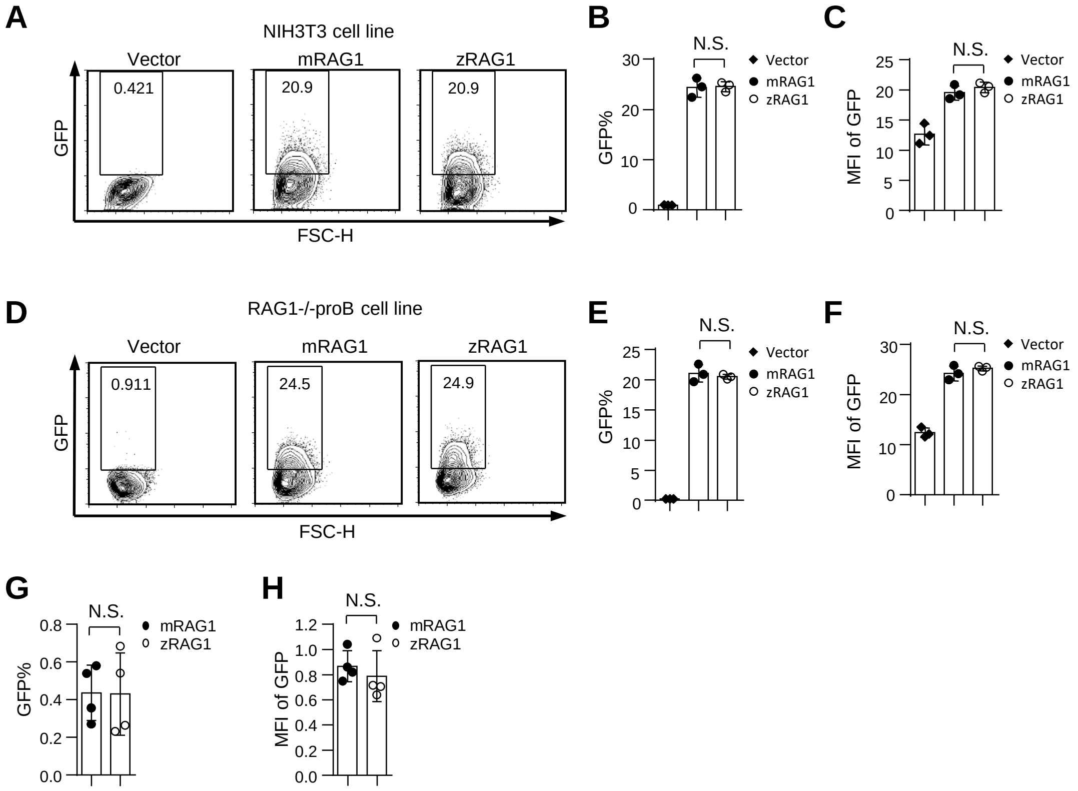

Figure S2. Both mRAG1 and zRAG1 have a similar stability. (A-C) and (D-F) zRAG1 protein showed a similar expression level compared to mRAG1 protein, and its MFI was also similar to mRAG1. zRAG1 protein-GFP and mRAG1-GFP were introduced into NIH3T3 cells (A-C) or RAG2-deficienct pro-B cells (D-F) by retrovirus-mediated gene transfer method. After 36 h, GFP expression and intensity was analyzed by FACS. (G and H) zRAG1 and mRAG1 have no difference in proteasome degradation. RAG1-deficient pro-B cells were treated with CHX (20 μg/ml) for 2 hours. GFP intensity was analyzed by FACS. Error bars indicate the SDs. The data are means ± standard deviation: $**P < 0.01$, $***P < 0.001$ by Student's t test; N.S.: no significance.

Figure S3

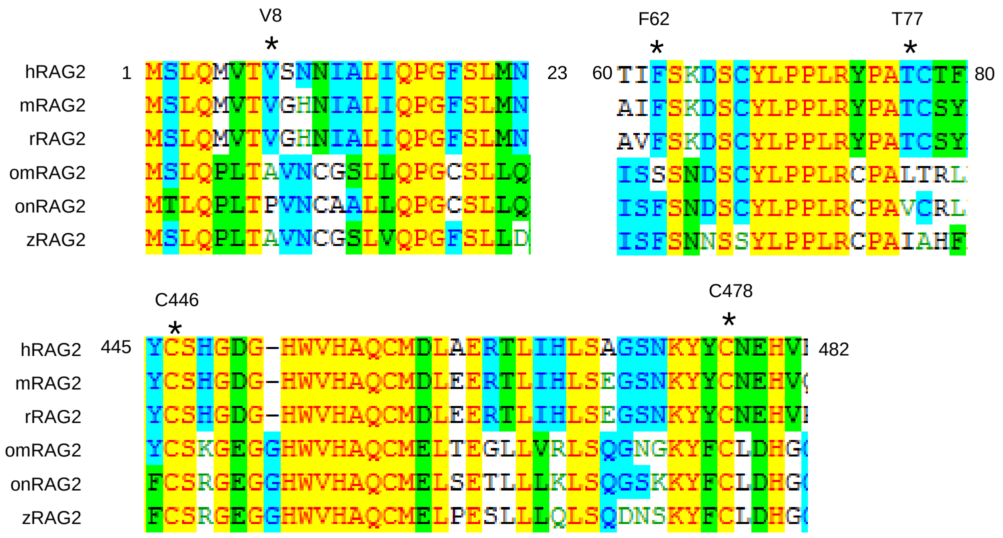

Figure S3. Comparison of amino acid sequence of ectotherms and endotherms. The results of the sequence alignment for three endotherms RAG2 (hRAG2, mRAG2 and rRAG2) and three ectotherms RAG2 proteins (omRAG2, onRAG2 and zRAG2) with Vector NTI 11 software. hRAG2, *Homo sapiens* RAG2; mRAG2, *Mus musculus* RAG2; rRAG2, *Rattus norvegicus* RAG2;omRAG2, *Oncorhynchus mykiss* RAG2; onRAG2, *Oreochromis niloticus* RAG2; zRAG2, *Danio rerio* RAG2. The asterisk represents the amino acid position that affect the stability of RAG2 or RAG1/RAG2 complex.

Figure S4

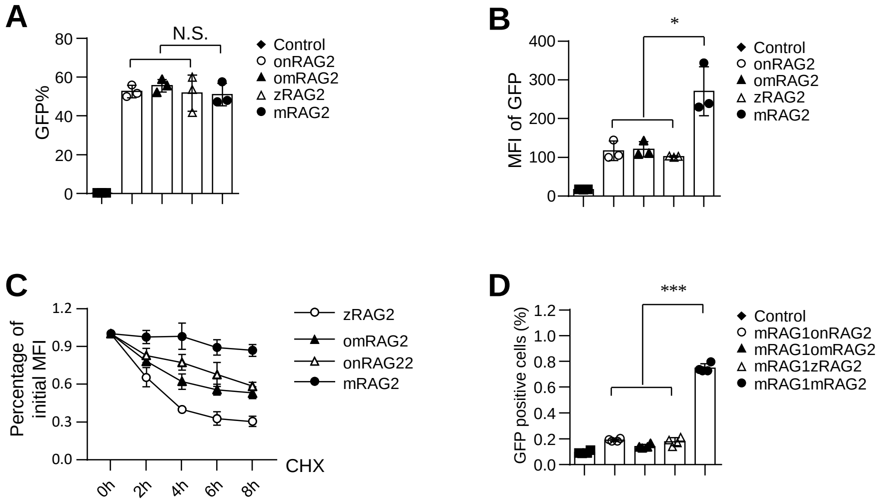

Figure S4. Comparison of RAG2 protein stability and recombination efficiency among three bony fish and mouse. (A and B) RAG2-GFP fusion proteins from nine species showed similar expression levels (A), but their MFIs (B) were different. After 36 h, GFP expression and intensity were analyzed by FACS. (C) RAG2-deficient pro-B cells were treated with CHX (20 μg/ml) for 8 hours (0-8h). The change of MFI of RAG2-GFP was shown. (D) NIH3T3 cells were co-transfected with pCJGFP (mouse), mRAG1 and RAG2 from four species using Lipo6000 transfection reagent. GFP intensity was analyzed by FACS. The error bars indicate the SDs. The data are presented as the mean ± standard deviation. $**P < 0.01$ and $***P < 0.001$ by Student's t test; N.S.: no significance. The species abbreviations are as follows: zRAG2, *Danio rerio* RAG2; on2, *Oreochromis niloticus* RAG2; omRAG2, *Oncorhynchus mykiss* RAG2; mRAG2, *Mus musculus* RAG2.

Figure S5

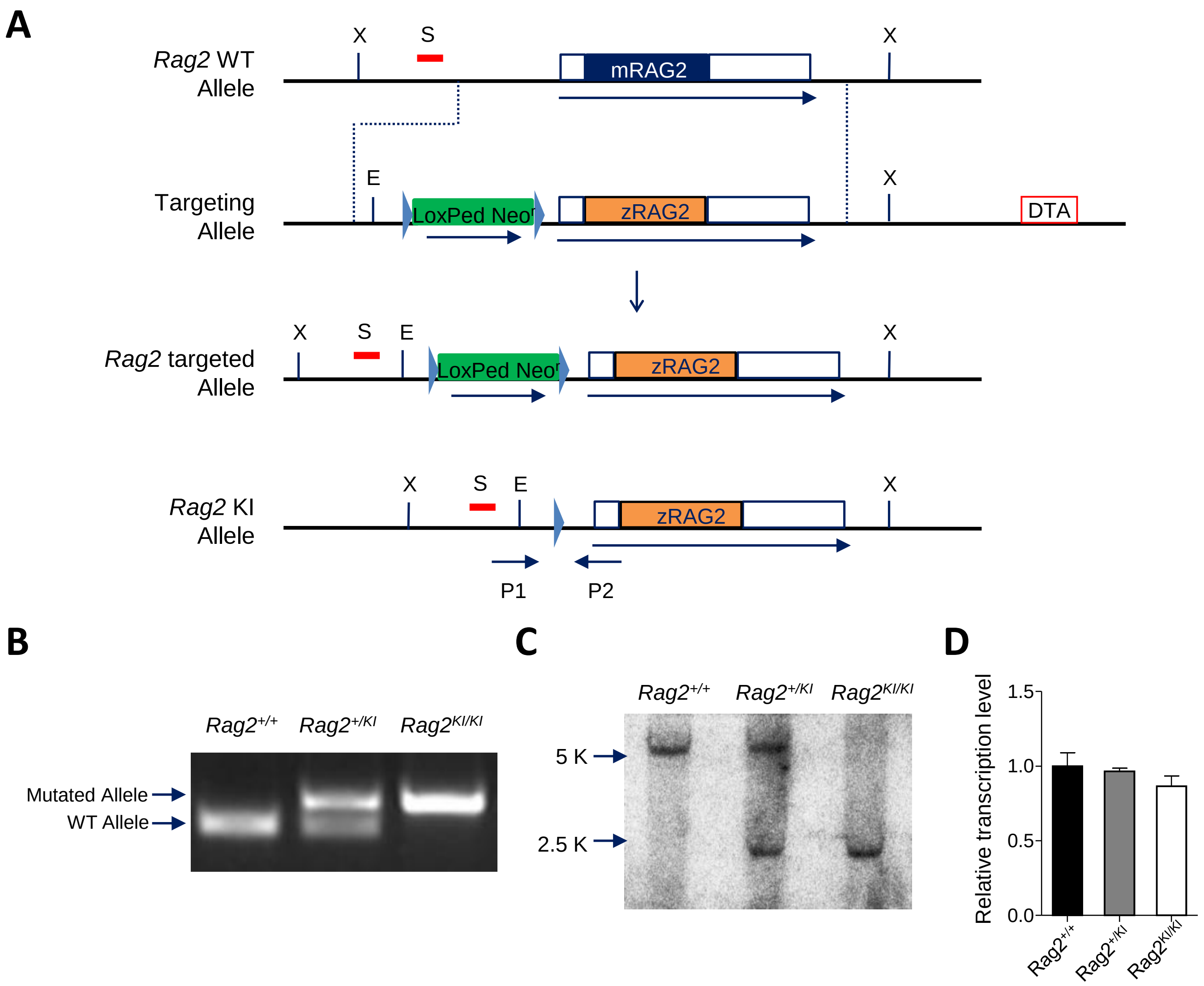

Figure S5. Generation of *Rag2*$^{KI/KI}$ mice. (A) A targeting vector was designed to substitute zebrafish *Rag2* for endogenous mouse *Rag2*. P1-P2, genotyping primers. S, southern probe. Restriction site: E, EcoRV; X, XbaI. (B) Tail DNA was extracted from *Rag2*$^{+/+}$, *Rag2*$^{+/KI}$ and *Rag2*$^{KI/KI}$ mice to confirm the removal of the floxed PgkNeo cassette by PgkCre. PCR analysis was performed with the primers indicated in A. (C) Southern blot analysis using the 5' probe on EcoRV and XbaI-digested genomic DNAs. The length of the fragment from wide type mice was 6 kb; the fragment from homozygous mice was 2.3 kb. Lane 1, *Rag2*$^{+/+}$ wild type mouse; lane 2, *Rag2*$^{+/KI}$ heterozygous mouse; lane 3, *Rag2*$^{KI/KI}$ homozygous mouse. (D) Analysis of transcription level of *Rag2*$^{+/+}$, *Rag2*$^{+/KI}$ and *Rag2*$^{KI/KI}$ mice by quantitative Real-time PCR.

Figure S6

A Comparison of $D_{\beta1}$-$J_{\beta1.1}$SJs from DN thymocytes

$D_{\beta1}$-$J_{\beta1.1}$	*Rag2*$^{+/+}$	*Rag2*$^{KI/KI}$
Precise joints	38/47 (80.9%)	41/50 (82%)
Imprecise joints	9/47 (19.1%)	9/50 (18%)
N nucleotides	9/47 (19.1%)	9/50 (19%)
Deletion	4/47 (8.5%)	0/50 (0%)

B Comparison of D_{δ}2-J_{δ}1 SJs from DN thymocytes

D_{δ}2-J_{δ}1	*Rag2*$^{+/+}$	*Rag2*$^{KI/KI}$
Precise joints	42/63(66.7%)	31/46(67.4%)
Imprecise joints	21/63(33.3%)	15/46(32.6%)
N nucleotides	20/21(95.2%)	15/15(100%)
Deletion	1/21(4.8%)	1/15(6.7%)

C Comparison of $D_{\beta2}$-$J_{\beta2}$ SJs from DN thymocytes

$D_{\beta2}$-$J_{\beta2.1}$	*Rag2*$^{+/+}$	*Rag2*$^{KI/KI}$
Precise joints	55/73(75.3%)	0/74(0%)***
Imprecise joints	18/73(24.7%)	74/74(100%)***
N nucleotides	18/18(100%)	74/74(100%)
Deletion	0/18(0%)	14/74(18.9%)

Figure S6. Comparison of recombination products between *Rag2*$^{KI/KI}$ and *Rag2*$^{+/+}$ mice. The number frequency and of each joint analyzed is shown. (A) Comparison of $D_{\beta1}$-$J_{\beta1.1}$ signal joints. (B) Comparison of D_{δ}2-J_{δ}1 signal joints. (C) Comparison of $D_{\beta2}$-$J_{\beta2.1}$ coding joints (mean ± SD; ***P < 0.001 by Student's t-test)

Figure S7

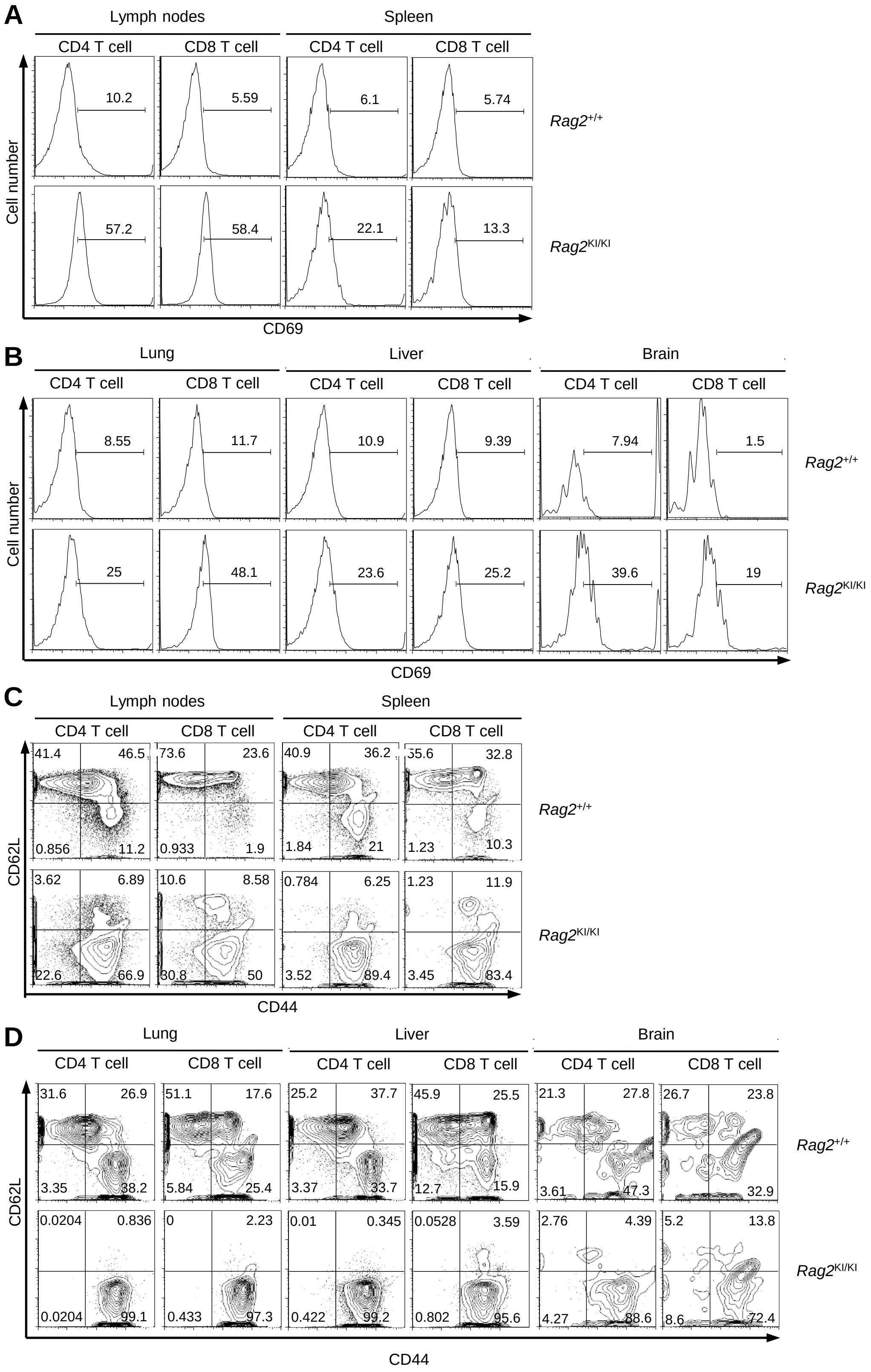

Figure S7. T cells of *Rag2*$^{KI/KI}$ mice exhibit a hyperactivated phenotype. CD4 and CD8 T cells from *Rag2*$^{KI/KI}$ and *Rag2*$^{+/+}$ mice were stained for the surface markers CD69 (A and B) and CD44 and CD62L (C and D) in the lymph nodes, spleen, lung, liver and brain and analyzed by flow cytometry.

Figure S8

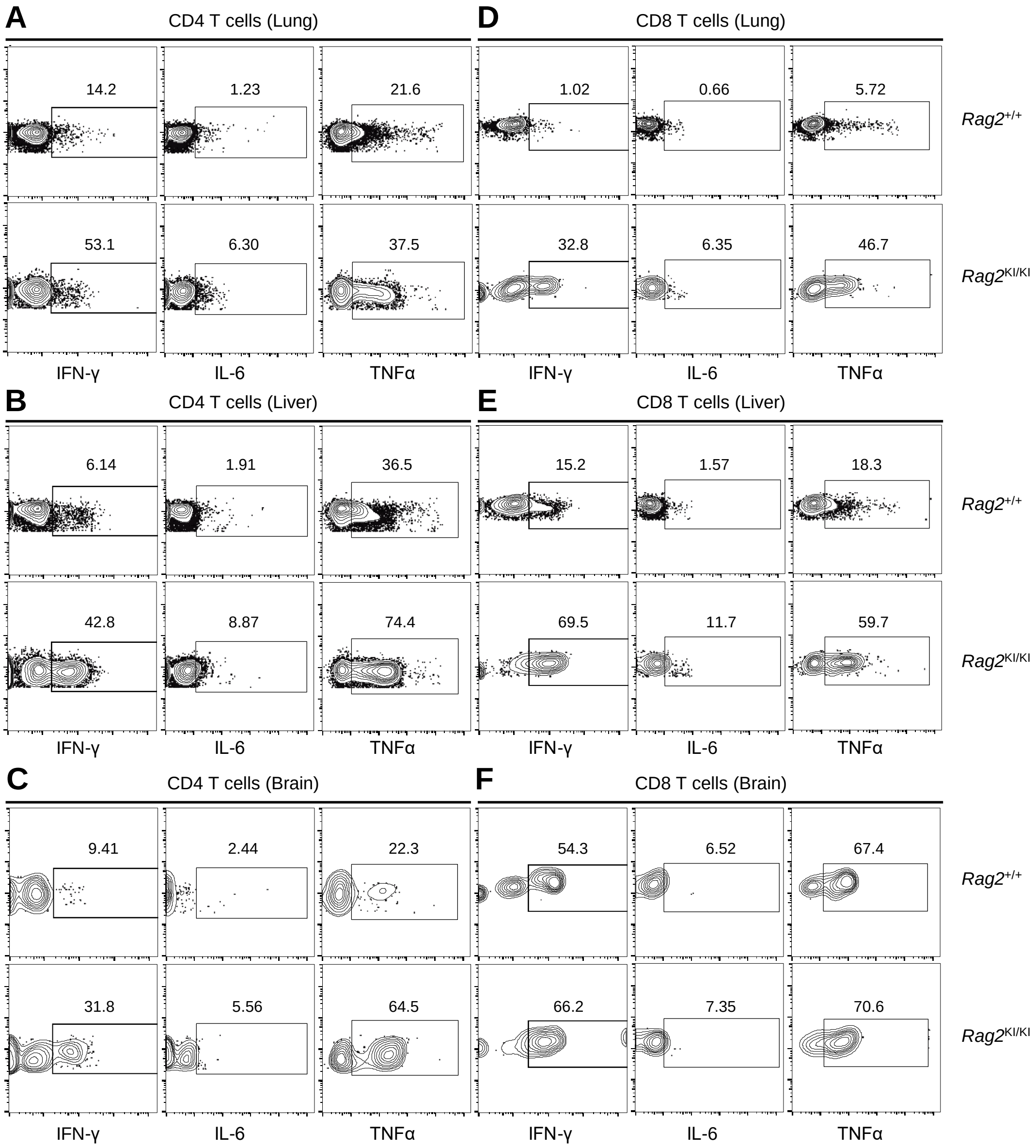

Figure S8. The increased percentage of cachexia cytokines IFN-γ, IL-6 and TNFα produced by CD4 and CD8 T cells of *Rag2*$^{KI/KI}$ mice. The increased percentage of cachexia cytokines IFN-γ, IL-6 and TNFα produced by CD4 T cells lung (A), liver (B) and brain (C) of *Rag2*$^{KI/KI}$ mice is analyzed. The increased percentage of cachexia cytokines IFN-γ, IL-6 and TNFα produced by CD8 T cells from lung (D), liver (E) and brain (F) of *Rag2*$^{KI/KI}$ mice is analyzed. All cytokines were detected by flow cytometric analysis after in vitro stimulation with PMA and ionomycin in the presence of brefeldin A for 4 h.
