# Supplementary material for: The evolution of zebrafish RAG2 protein is required for adapting to the elevated body temperature of the higher endothermic vertebrates
Source: Sci Rep. 2020 Mar 5;10:4126. doi: 10.1038/s41598-020-61019-w (PMC7057966; doi:10.1038/s41598-020-61019-w)

Supplementary information - Raw data

The evolution of zebrafish RAG2 protein is required for adapting to the elevated body temperature of the higher endothermic vertebrates

Ao Sun[1,+], Ke Xu[1,+], Haifeng Liu[1], Hua Li[1], Yaohuang Shi[1,2], Xiaoyan Zhu[1], Tao Liang[1], Xinyue Li[1], Xianxia Cao[1,2], Yanhong Ji[3], Taijiao Jiang[4], Chenqi Xu[1] and Xiaolong Liu[1,2,*]

[1]State Key Laboratory of Cell Biology, Shanghai Institute of Biochemistry and Cell Biology, Center for Excellence in Molecular Cell Science, Chinese Academy of Sciences, Shanghai 200031; University of Chinese Academy of Sciences, China

[2]School of Life Science and Technology, ShanghaiTech University, Shanghai 201210, China;

[3]Department of Pathogenic Biology and Immunology, School of Basic Medical Sciences, Xi'an Jiaotong University Health Science Centre, Xi'an, Shaanxi, 710061, China;

[4]Center of System Medicine, Institute of Basic Medical Sciences, Chinese Academy of Medical Sciences & Peking Union Medical College, Beijing, 100005, China

[*]Correspondence should be addressed to X.L. (liux@sibcb.ac.cn)

[+]A.S. and K.X. contributed equally to this work.

Keywords: RAG2, stability, temperature, evolution, jawed vertebrates, cachexia

Figure 1I

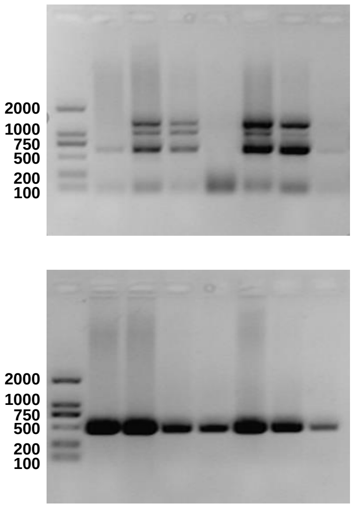

Figure 1J

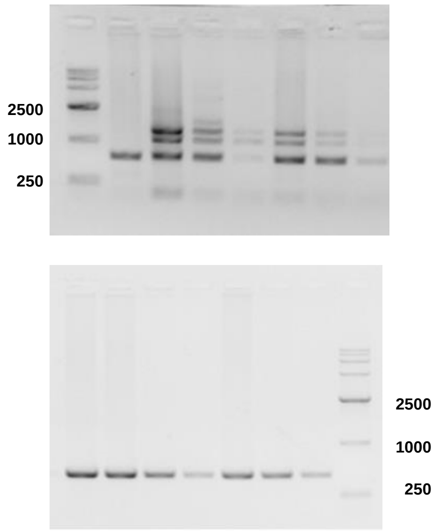

Figure 2B

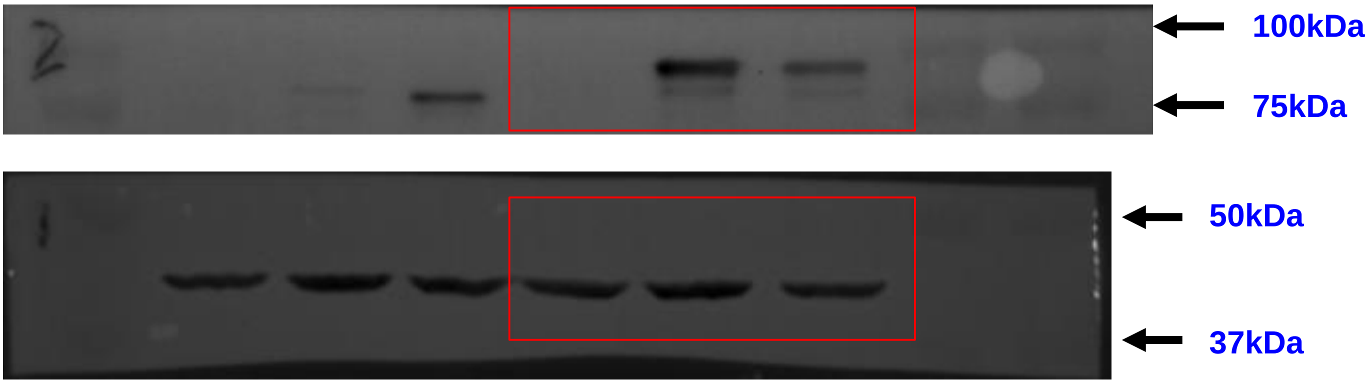

Figure 3J

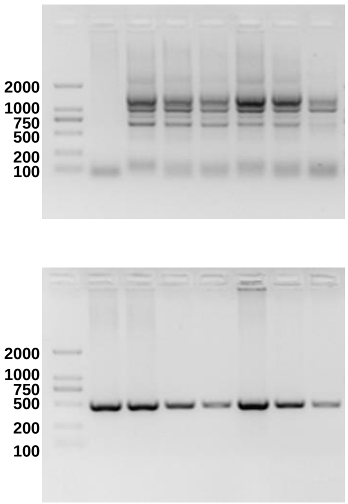

Figure 4A

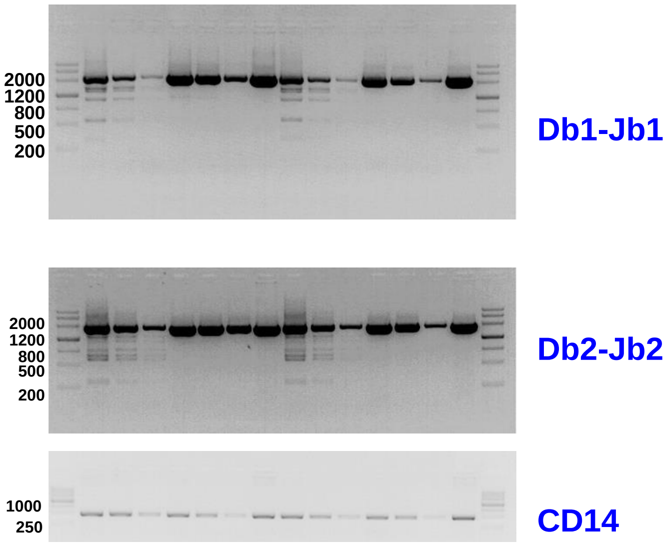

Figure 4B

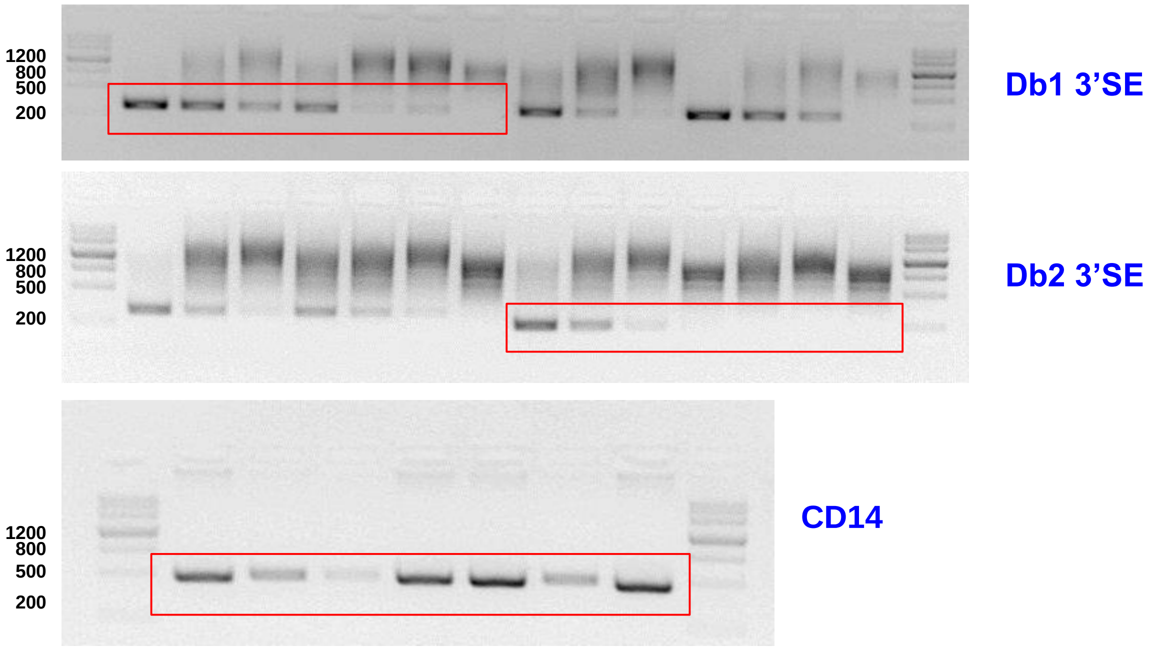

Figure S1E

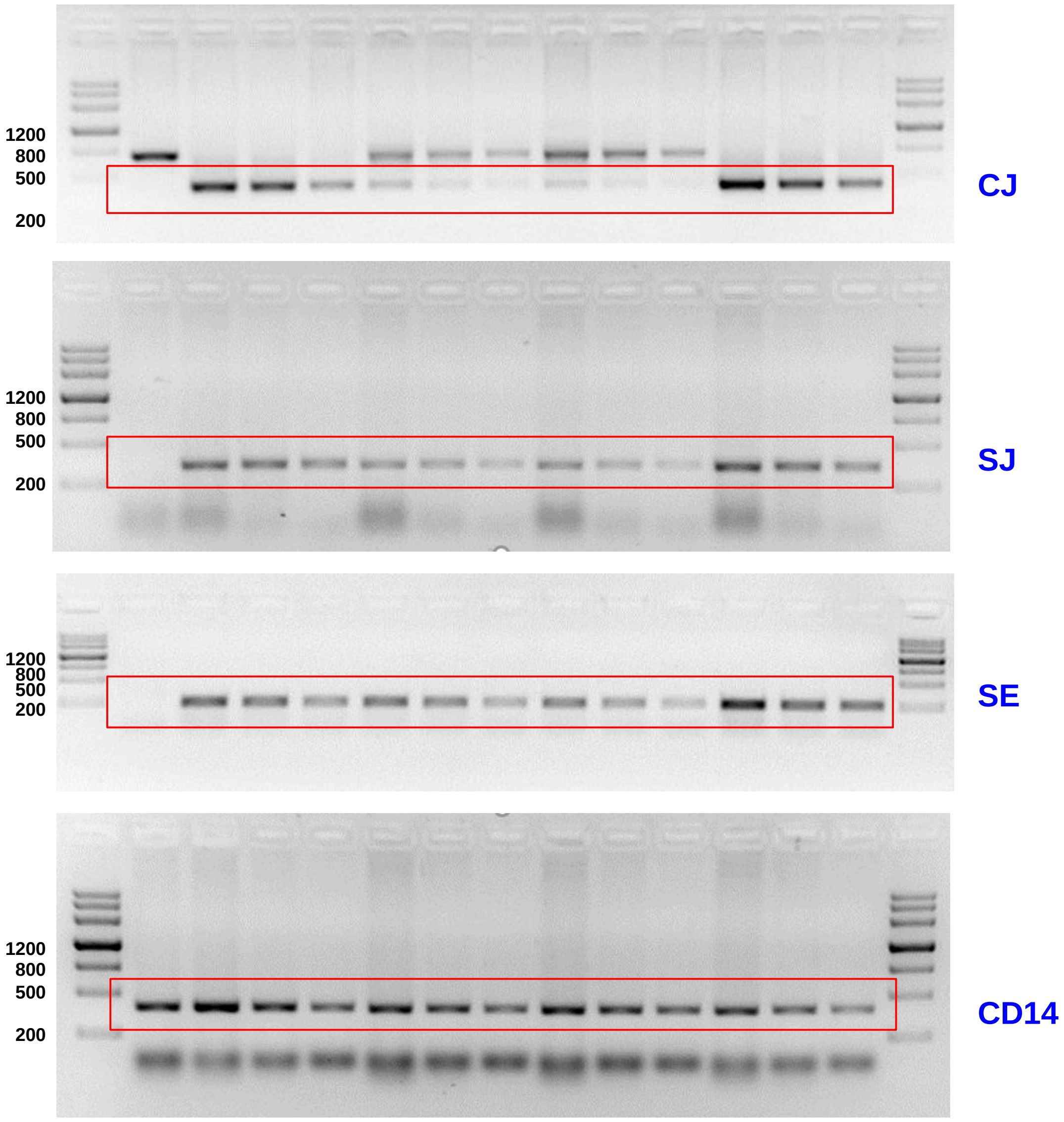

Supplement: Supplementary file 2 — Supplementary information 2 [file 41598_2020_61019_MOESM2_ESM.pdf]
